# Exploring Tumor Cell–Platelet Biochemical Interactions by Dielectric Measurements of Blood: A Potential Target for Tumor Detection and Staging

**DOI:** 10.3390/biology14050542

**Published:** 2025-05-13

**Authors:** Annamaria Russo, Ester Tellone, Francesco Farsaci

**Affiliations:** Department of Chemical, Biological, Pharmaceutical and Environmental Sciences, University of Messina, Viale Ferdinando Stagno d’Alcontres 31, 98166 Messina, Italy; etellone@unime.it (E.T.); farsaci@ipcf.cnr.it (F.F.)

**Keywords:** non-equilibrium thermodynamics, cancer, circulating tumor cells, platelets, tumor staging

## Abstract

This paper aims to investigate the dielectric properties of blood for tumor detection and staging. The application of complex thermodynamic models and the study of the trend over time of some thermodynamic functions have allowed us to highlight the generation of displacement currents caused by changes in charge, i.e., by the activation and consequent accumulation of platelets on migrating tumor cells. Although few studies exist to date in this regard, the technique used has provided promising results, especially in terms of building a database. In this context, the evaluation of the dielectric parameters of healthy and cancerous blood can be exploited for the staging of cancer. The main advantages of this method include easy application, non-invasiveness, low cost, and online monitoring.

## Simple Summary

Cancer is one of the leading causes of death worldwide, so it is imperative to understand the signaling pathways that drive tumor progression and translate knowledge into better treatment strategies. The study of neoplastic processes through thermodynamic procedures, using sophisticated mathematical models, opens new avenues, highlighting evolutionary and characterizing details otherwise impossible to evaluate. Research characterizing the dielectric properties of cancer tissues is still limited, but it is already known that changes within and around cancer cells in the bloodstream strongly influence these properties. Thus, the present theoretical investigation focuses on the difference in blood dielectric properties between healthy rat models and those with lung cancer, monitoring tumor development at different time points. The effects of neoplastic phenomena were calculated with the dielectric relaxation technique developed with the non-equilibrium thermodynamic approach with internal variables. This study analyzed for the first time the role of displacement currents to explain otherwise unexplainable phenomena, i.e., the decrease over time of cancer cells in the blood. Furthermore, dielectric analysis allowed us to determine the peculiar characteristics of the intravasation phenomenon that evolves in the blood during cancer pathology, and it could lay the groundwork for a new diagnostic approach.

## 1. Introduction

Cancer is a leading cause of death worldwide. Despite advances in chemo- and radiotherapy, and surgery, many types of cancer continue to have a low five-year survival rate. Therefore, there is a need to better understand the molecular signaling pathways driving tumor formation and progression, and to translate that knowledge into improved therapeutic strategies [1]. Mouse models are often used for studying tumorigenesis, monitoring tumor growth, and testing novel therapeutic approaches. Since mouse models possess clinical features of advanced human cancer, they are turning out to be a useful tool for translational cancer research [2]. Two types of xenograft animal cancer models are available for researchers: ectopic and orthotopic animal models. In an ectopic cancer model, the tumor is subcutaneously implanted, whereas in an orthotopic cancer model, cancer tissues are implanted in the analogous or orthotopic sites in the animals. The latter is generally preferred because the ectopic cancer model does not adequately represent cancer since the microenvironment plays a crucial role in cancer development [3]. Orthotopic murine models allow the investigation of cancer formation time and the life span of the animals following transplantation/inoculation. Cells are monitored for progression, invasion, and metastasis at different time points of tumor development. It is noteworthy that the tumor models used in most preclinical studies are very fast-growing [4]. The literature data reported that the tumorigenicity time (i.e., the time from the inoculation to the formation of a palpable tumor) is generally 7–15 days after inoculation, which means that the tumor cell propagation occurred rapidly, with solid mass formations after 10–15 days of inoculation [5]. The spread of cancer cells from the primary tumor to surrounding tissues and other body districts occurs through the blood (intra- and extravasation) and involves the generation of Circulating Tumor Cells (CTCs) [6], which are shed from solid cancers in the form of single or clustered cells that are often referred to as Circulating Tumor Microemboli (CTMs), with the latter displaying a good ability to initiate metastasis [7]. After entering the blood circulation, CTCs become exposed to various blood cells, such as platelets, neutrophils, monocytes, macrophages, and endothelial cells, which may alter the behavior of CTCs themselves, playing concerted pro-metastatic roles [8]. Thrombocytosis is frequently observed in metastatic patients, suggesting the crucial role of platelets in metastasis, besides their well-known role in hemostasis and coagulation. The degree of tumor engraftment, growth rate, timing, and frequency of metastases in animal models are dependent on the cell line employed for inoculation, as rightly reported by Justilien et al. [2]. As an example, Olofsson et al. compared the speed of tumor growth of different cell lines: B16-F10 tumors (melanoma cells) grow out in 10–14 days, whereas 4T1 breast tumors are a little slower, taking approximately 21 days [4]. As cancer research may benefit from the use of experimental animal models that closely mimic the clinical situation, several studies have been published providing methods for in vivo imaging and monitoring of tumor growth. Lung orthotopic tumors produced by inoculation of H1299 cells transfected to express luciferase were monitored in vivo for progression, invasion, and metastasis using an IVIS (in vivo imaging system) [2]. Approximately one week after implantation, an initial focus at the injection site in the lung was visualized, followed by local growth of the primary tumor. Approximately four weeks after implantation, metastatic spread to the lungs and the lymph nodes became visible. Tumor-bearing mice survived for approximately seven weeks, succumbing to thoracic metastasis [2]. An orthotopic animal model of lung cancer was built up through transplanting A549 cells into the lungs of nude mice with the aim of evaluating the growth pattern of intrathoracic tumors by spiral Computerized Tomography (CT). Small lung nodules were observed one week after intrathoracic injection of tumor cell suspension and gradual growth of the intrathoracic tumor mass over time [9]. Male Wistar rats inoculated with Walker 256 breast carcinoma have been monitored at different times of the tumor development (12 h, 24 h, 48 h, 3, 5, 7, 10, and 14 days), pointing out that the tumor grew exponentially within 14 days, reaching an average of 2% of the animal weight. After 14 days of subcutaneous injection of the cells, a solid tumor was observed. At this time, the protocol was ended to avoid cachexia and further unnecessary animal suffering [5]. The current clinical diagnostic methods for carcinoma include CT, Magnetic Resonance Imaging (MRI), Positron Emission Tomography (PET) scan, ultrasound, and X-ray, among others. To the best of our knowledge, only a few investigations have proposed the use of blood dielectric measurements for carcinoma recognition so far [10,11,12,13]. As the electrical features of cells are affected by the microenvironment, detecting changes in the dielectric properties of tissues can provide simple and cost-effective tools for cancer detection and monitoring. The changes within and around cancer cells in the bloodstream strongly influence dielectric properties. Thus, the evaluation of dielectric parameters of the healthy and cancerous blood, reflecting the functional state of cells, can be exploited for the detection of malignant tissues and for cancer staging. The main advantages of this method include easy application, non-invasiveness, low cost, and online monitoring. Nevertheless, to the best of our knowledge, studies characterizing the dielectric properties of cancer tissues are limited. This paper proposes a reproducible, easy-to-establish dielectric model for monitoring tumor progression. This technique can be used in early tumor detection based on the difference in the dielectric properties between normal and malignant tissues.

## 2. Materials and Methods

The present theoretical investigation was inspired by the study of Chen et al. [14] that focused on the difference in blood dielectric properties between normal and lung carcinoma rats, monitoring tumor development at different time points. Briefly, a Lewis lung carcinoma cell suspension was inoculated into rats outside the right forelimb axillaries to produce a lung tumor model. Orbital blood and tail artery blood were collected to perform dielectric spectroscopy measurements at 7, 14, 21, 28, and 32 days.

### 2.1. Thermodynamic Scheme

Non-equilibrium thermodynamics is one of the most important disciplines for the study of both physiological and pathological biological phenomena. In particular, the introduction of the so-called internal variables allows the study of evolutionary and characterizing details that would otherwise be impossible to evaluate. Referring to dielectric relaxation phenomena, this branch of physics, combined with experimental approaches, allows the determination of the frequency spectra of some coefficients and functions with specific physical meanings, as we will see. Starting from the production of entropy expressed by internal variables, the decomposition of the polarization vector into two parts is obtained: one associated with polarization by strain and one associated with polarization by orientation. The latter phenomenon is very present in biological molecules when subjected to sinusoidal electric fields. Using particular thermodynamic procedures, other functions are introduced whose physical significance opens new avenues for the study of particular neoplastic processes, highlighting new aspects.

### 2.2. Kluitenberg’s Non-Equilibrium Thermodynamic Synthesis

Generally, for the thermodynamic study of a dielectric medium, the specific entropy S is considered as a function of the specific Internal Energy, U, and the specific polarization, P, which is as follows:

S = S(U|P) (1)

Gutenberg’s theory [15,16,17] is based on the idea that these variables are insufficient to describe dielectric relaxation phenomena, thus introducing a new variable P^(1)^ so that the vector P can be decomposed into two parts, P^(0)^ and P^(1)^, as follows:

P = P^(0)^ + P^(1)^ (2)

where, following the Debye model, P^(0)^ is associated with the polarization by the formation and P^(1)^ with the polarization by orientation [15,16,17]. We will therefore have the following:

S = S(U|P, P^(1)^) (3)

Based on the latter, the following functions are introduced:

$$\left\{\begin{array}{ll} {\underline{E}}^{\left(eq\right)}=-T\frac{\partial s}{\partial \underline{P}} & \;(4)\\ {\underline{E}}^{\left(ir\right)}=\underline{E}-{\underline{E}}^{\left(eq\right)} & \;(5)\\ {\underline{E}}^{\left(1\right)}=-T\frac{\partial s}{\partial {\underline{P}}^{\left(1\right)}} & \;(6) \end{array}\right.$$

where E^(eq)^ is the equilibrium electric field vector, E^(ir)^ is the irreversible electric field vector, E = E^(eq)^ + E^(ir)^ are the electric field vectors that appear in Maxwell’s equations, E^(1)^ is the electric field vector associated by the change in specific entropy S with respect to P^(1)^, and T is the temperature constant assumed as it really happens in biological phenomena for sufficiently small intervals of time. The mathematical description of extremely complex biological phenomena, such as tumors, when observed in detail, imposes a non-linearity in the equations that describe them. However, linear models can be introduced that, in some cases, even if they do not provide rigorous information, are of great help in early diagnosis and in the almost univocal characterization of neoplastic phenomena. It is clear that the more independent phenomena characterize or describe a phenomenon, the more singular its detectability will be. The linear response theory is well suited for such a purpose. Equations (4) and (5) have a strongly non-linear form and are demonstrated by linear response theory, which can be linearized [15,16,17], obtaining the following equations:

(7) $${\underline{E}}^{\left(\mathrm{eq}\right)}=a^{\left(0,0\right)}\;{\underline{P}}^{\left(0\right)}$$

(8) $${\underline{E}}^{\left(1\right)}=E^{\left(\mathrm{eq}\right)}+(a^{\left(0,0\right)}-a^{\left(1,1\right)})\;{\underline{P}}^{\left(1\right)}$$

These equations are called State Equations, and the coefficients a^(0,0)^ and a^(1,1)^ are State Coefficients and are related to elastic and inelastic processes, respectively.

They have the dimensions of the reciprocal of the dielectric constant ε:

(9) $$[a^{\left(0,0\right)}]=[a^{\left(1,1\right)}]=\frac{1}{\left[\varepsilon \right]}$$

The entropy production was determined based on (3) as follows:

(10) $$\sigma \left(S\right)=T^{-1}\;\left({\underline{E}}^{\left(ir\right)}\frac{dP}{dt}+{\underline{E}}^{\left(1\right)}\frac{dP^{\left(1\right)}}{dt}\right)$$

And using a standardized method developed by De Groot and Mazur [18], the following phenomenological equations are obtained (where cross effects are neglected):

(11) $$E^{\left(\mathrm{ir}\right)}=L^{\left(0,0\right)}\frac{dP}{dt}$$

(12) $$\frac{dP^{\left(1\right)}}{dt}=L^{\left(1,1\right)}E^{\left(1\right)}$$

The L^(0,0)^ and L^(1,1)^ coefficients are called phenomenological coefficients and have the dimensions of a resistance and a conductivity, respectively. L^(0,0)^ is connected to irreversible processes related to the change in P; L^(1,1)^ is related to the change in P^(1)^ and the corresponding intensive variable E^(1)^. Both are related to irreversible change in polarization and not to conservative processes. Essentially, these coefficients describe how different physical processes interact within a system, based on phenomenological (observable) behavior, rather than deeper theoretical derivations. These coefficients are used in various fields, such as thermodynamics or transport theory, to model and understand systems without needing microscopic details [19,20,21,22]. The four coefficients, a^(0,0)^, a^(1,1)^, L^(0,0)^, and L^(1,1)^, do not depend on time but can depend on a parameter which, as we will see, is the frequency. This frequency dependence means that their response may change based on the speed of the applied stimuli. Next to field E^(1)^ is field E^(1)^_P_, which is obtained as follows:

(13) $${\underline{E}}_{\underline{P}}^{\left(1\right)}=(a^{\left(0,0\right)}-a^{\left(1,1\right)})\;{\underline{P}}^{\left(1\right)}$$

and is associated with the P^(1)^ polarization.

By manipulating Equations (7), (8), (11) and (12), the so-called differential equation of dielectric relaxations is obtained:

(14) $$\chi_{EP}^{\left(0\right)}\underline{E}+\frac{dE}{dt}=\chi_{PE}^{\left(0\right)}\;\underline{P}+\chi_{PE}^{\left(1\right)}\frac{dP}{dt}+\chi_{PE}^{\left(2\right)}\;\frac{d^{2}P}{dt^{2}}$$

where

$$\left\{\begin{array}{ll} \chi_{EP}^{\left(0\right)}=a^{\left(1,1\right)}L^{\left(1,1\right)} & \;(15)\\ \chi_{PE}^{\left(0\right)}=a^{\left(00\right)}\;\left(a^{\left(1,1\right)}-a^{\left(0,0\right)}\right)\;L^{\left(1,1\right)} & \;(16)\\ \chi_{PE}^{\left(1\right)}=a^{\left(0,0\right)}+a^{\left(1,1\right)}L^{\left(0,0\right)}L^{\left(1,1\right)} & \;(17)\\ \chi_{PE}^{\left(2\right)}=L^{\left(0,0\right)} & \;(18) \end{array}\right.$$

### 2.3. Dielectric Relaxations

We will only deal with one-dimensional problems so as to consider only scalar quantities. The study of dielectric relaxation phenomena is based on the idea that a material system is stimulated by an extensive quantity (input, cause), which in our case will be polarization

(19) $$P=P_{0}sen\;\omega t$$

since ω is the frequency of stress and the response is observed, which is an intensive quantity (output, effect), as follows:

(20) $$E=E_{0}\left(\omega \right)\mathrm{sen}\;[\omega t\;+\Phi (\omega )]$$

where E_0_(ω) is the amplitude of the electric field; Φ(ω) is the phase lag between P and E. Γ1(ω) and Γ2(ω) indicate the storage and loss dielectric moduli relating to non-dissipative and dissipative phenomena, respectively, which is obtained from (19) and (20):

$$\left\{\begin{array}{ll} \Gamma_{1}\left(\omega \right)=\frac{E_{0}\left(\omega \right)}{P_{0}}\cos\Phi \left(\omega \right) & \;(21)\\ \Gamma_{2}\left(\omega \right)=\frac{E_{0}\left(\omega \right)}{P_{0}}\;\mathrm{sen}\;\Phi \left(\omega \right) & \;(22) \end{array}\right.$$

The integration of differential Equation (14), where (19) is taken into account, and the comparison with (20) taking into account (21) and (22), allows us to write a very useful expression for our purposes of the four coefficients, a^(0,0)^, a^(1,1)^, L^(0,0)^ and L^(1,1)^, as a function of ω:

$$\left\{\begin{array}{ll} a^{\left(0,0\right)}=\Gamma_{1}+\frac{\Gamma_{2}^{\left(1\right)}}{\omega \delta } & \;(23)\\ a^{\left(1,1\right)}=\frac{{\left[\Gamma_{2}^{\left(1\right)}+\Gamma_{1}\omega \delta \right]}^{2}}{\omega \delta \Gamma_{2}^{\left(1\right)}(1+\omega^{2}\delta^{2})} & \;(24)\\ L^{\left(1,1\right)}=\frac{\omega \Gamma_{2}^{\left(1\right)}\left(1+\omega^{2}\delta^{2}\right)}{{\left[\Gamma_{2}^{\left(1\right)}+\Gamma_{1}\omega \delta \right]}^{2}} & \;(25)\\ L^{\left(0,0\right)}=\frac{\Gamma_{2R}}{\omega } & \;(26) \end{array}\right.$$

where δ is the relaxation time

(27) $$\Gamma_{2}^{\left(1\right)}=\Gamma_{2}-\omega L^{\left(0,0\right)}$$

and $\Gamma_{2R}$ is the relaxed value of $\Gamma_{2}$. We want to remember that there is the following link between $\Gamma_{1}^{\left(\omega \right)}$ and $\Gamma_{2}^{\left(\omega \right)}$ and the complex dielectric constant $\varepsilon_{\left(\omega \right)\;}$

(28) $$\Gamma_{1}=\frac{\varepsilon_{1}-\varepsilon_{0}}{{\left(\varepsilon_{1}-\varepsilon_{0}\right)}^{2}+\varepsilon_{2}^{2}}$$

(29) $$\Gamma_{2}=\frac{\varepsilon_{2}}{{\left(\varepsilon_{1}-\varepsilon_{0}\right)}^{2}+\varepsilon_{2}^{2}}$$

where $\varepsilon_{0}$ is the dielectric constant of vacuum and $\varepsilon_{1}$ and $\varepsilon_{2}$ are the real and imaginary parts of $\varepsilon_{\left(\omega \right)}$. In summary, we introduced the following 12 functions:

(30) $${\underline{P}}^{\left(0\right)},\;{\underline{P}}^{\left(1\right)},\;{\underline{E}}^{\left(\mathrm{eq}\right)},\;{\underline{E}}^{\left(1\right)},\;{\underline{E_{P}}}^{\left(1\right)},\;{\underline{E}}^{\left(\mathrm{ir}\right)},\;\frac{d{\underline{P}}^{\left(1\right)}}{dt},\;a^{\left(0,0\right)},\;a^{\left(1,1\right)},\;L^{\left(0,0\right)},\;L^{\left(1,1\right)},\;\sigma^{\left(s\right)}$$

Based on Equations (2), (7), (8), and (11)–(13), the following equations are obtained [19]:

(31) $${\underline{E}}^{\left(\mathrm{ir}\right)}=L^{\left(0,0\right)}\frac{dP}{dt}=\Gamma_{2R}P_{0}\omega \;\cos\;\omega t$$

(32) $${\underline{E}}^{\left(\mathrm{eq}\right)}=\Gamma_{1}P$$

(33) $${\underline{P}}^{\left(0\right)}\;\;\frac{{\underline{E}}^{\left(eq\right)}}{a^{\left(0,0\right)}}$$

(34) $${{\underline{E}}_{\underline{P}}}^{\left(1\right)}\;\left(a^{\left(0,0\right)}-a^{\left(1,1\right)}\right)\;{\underline{P}}^{\left(1\right)}$$

(35) $$\frac{d{\underline{P}}^{\left(1\right)}}{dt}=P_{0}\;\omega \;\cos\;\omega t\left(1-\frac{\Gamma_{1}}{a^{\left(0,0\right)}}\right)$$

(36) $${\underline{P}}^{\left(1\right)}=P\;\left(1-\frac{\Gamma_{1}}{a^{\left(0,0\right)}}\right)$$

(37) $${\underline{E}}^{\left(1\right)}=\frac{1}{L^{\left(1,1\right)}}P_{0}\omega \;\cos\;\omega t\left(1-\frac{\Gamma_{1}}{a^{\left(0,0\right)}}\right)$$

(38) $$\sigma^{\left(s\right)}=\Gamma^{-1}P_{0}^{2}\;\left[\omega \Gamma_{2R}+\frac{\omega^{2}}{L^{\left(1,1\right)}}\;\left(1-\frac{\Gamma_{1}}{a^{\left(0,0\right)}}\right)\right]\;\cos^{2}\;\omega t$$

If a perturbed biological system is correlated with a harmonic electric field of frequency ω and the experimentally obtained complex dielectric constant, the 12 sizes of Equation (30) can be calculated. These functions identify a phenomenon occurring in the system and, therefore, the system itself. Based on Equation (30), it is possible to write the matrix named Thermodynamic Tumor Matrix (TTM):

(39) $$\mathrm{TTM}=\left\|\begin{array}{llll} a^{\left(0,0\right)} & a^{\left(1,1\right)} & L^{\left(0,0\right)} & L^{\left(1,1\right)}\\ {\underline{P}}^{\left(0\right)} & {\underline{P}}^{\left(1\right)} & {\underline{E}}^{\left(1\right)} & {\underline{E}}_{\underline{P}}^{\left(1\right)}\\ {\underline{E}}^{\left(eq\right)} & {\underline{E}}^{\left(ir\right)} & \frac{d{\underline{P}}^{\left(1\right)}}{dt} & \underline{\sigma }(s) \end{array}\right\|$$

TTM thermodynamically analyzes the tumor cells compared to physiological cells and their interactions with the surrounding environment (in this case, the blood). The knowledge provided from the functions present in this matrix is more selective and detailed than ε′ and ε″, which will better characterize the tumor. ε′ and ε″ give the “sum” of all dissipative phenomena and do not specify their origins; as a result, it is not possible to know how events change in relation to each other. Since the functions of the TTM can show differences, biological tissues can be studied, contributing to a more precise characterization of the tumor.

### 2.4. Mathematical Approach

Recalling the following equations [19,20,21,22]:

(40) $$a^{\left(0,0\right)}={\;\Gamma }_{1}+\frac{\Gamma_{2}^{\left(1\right)}}{\omega \sigma }$$

(41) $$a^{\left(1,1\right)}=\frac{{(\Gamma }_{1}\omega \sigma +\Gamma_{2}^{\left(1\right)}{)}^{2}}{\omega \sigma \Gamma_{2}^{\left(1\right)}(1+\omega^{2}\sigma^{2})}$$

trivially, the result is as follows:

(42) $$a^{\left(0,0\right)}-a^{\left(1,1\right)}=\frac{\Gamma_{1}\omega \sigma +\Gamma_{2}^{\left(1\right)}}{\Gamma_{2}^{\left(1\right)}(1+\omega^{2}\sigma^{2})}\left(\Gamma_{2}^{\left(1\right)}\omega \sigma -\Gamma_{1}\right)$$

This produces a result of a^(0,0)^ = a^(1,1)^ if the following are satisfied:

(43) $$\Gamma_{1}{=\Gamma }_{2}^{\left(1\right)}\omega \sigma$$

Substituting the latter in Equations (40) and (41), we will obtain the following result:

(44) $$a^{\left(0,0\right)}=a^{\left(1,1\right)}=\Gamma_{2}^{\left(1\right)}(\frac{1+\omega^{2}\sigma^{2}}{\omega \sigma })$$

Obviously, Equation (43) is not satisfied for any ω values, but it should be resolved with respect to ω if the analytic expression of $\Gamma_{1}$ and ${\;\Gamma }_{2}^{\left(1\right)}={(\Gamma }_{2}-\Gamma_{2R})$ is known, where $\Gamma_{2R}$ is the relaxed value of $\Gamma_{2}$. Thus, the theoretical ω values, for which Equation (43) is valid, will be found. These values will be finite, and it is possible to indicate them with ω_i_, where *i* varies between 0 and the number of measurements made. That is, each ω_i_ referred to the *i*-th measure is the value for which Equation (43) is valid. In this case, “*i*” can assume values 0, 1 … 6 because 6 are the measurements made. Hence, Equation (43) should be written as follows:

(45) $$\Gamma_{1}{(\omega_{i})=\Gamma }_{2}^{\left(1\right)}(\omega_{i})\omega_{i}\sigma$$

As we shall see, the latter allows us to establish, with an approximation depending on the number of measures carried out, how long the invasion began and the consequences that arise from it. Let us see the determination of some thermodynamic functions when condition (43) is imposed. Since the presence of permanent dipoles prevails in the sample, it will refer only to 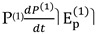 as they are related to polarization by orientation. Let us start with the determination of P^(1)^ by recalling the following [22]:

(46) $$P^{\left(1\right)}=P(1-\frac{\Gamma_{1}}{a^{\left(0,0\right)}})$$

Substituting Equations (43) and (44), we obtain the following:

(47) $$P^{\left(1\right)}=P(\frac{1}{1+\omega^{2}\sigma^{2}})$$

In the latter equation, knowing σ and substituting the ω value that satisfies Equation (43), all the values of P^(1)^ will be found at different times for which a^(0,0)^ = a^(1,1)^. Recalling that one of these ω (and therefore a time t) is the separator element between two or more biological phenomena, we aim to evaluate these phenomena. Obviously, to determine the ω value that satisfies Equation (43), one should know the analytic expression of ${\;\Gamma }_{1}$ and $\Gamma_{2}$. This latter value can be determined from the experimental data by selecting the ω values at which the curves a^(0,0)^ and a^(1,1)^ cross. To solve Equation (43), the Debye model, from which ${\;\Gamma }_{1}$ and $\Gamma_{2}$ were obtained, was used as follows:

(48) $${\;\Gamma }_{1}=\frac{{(a+bx}^{2})(1+x^{2})}{b^{2}{x^{4}+x}^{2}\left(a^{2}+b^{2}\right)+a^{2}}$$

(49) $${\;\Gamma }_{2}=\frac{x(a-b)(1+x^{2})}{b^{2}{x^{4}+x}^{2}\left(a^{2}+b^{2}\right)+a^{2}}$$

where

x = ωσ(50)

(51) $$a=\varepsilon_{1R}-1$$

(52) $$b=\varepsilon_{1U}-1$$

$\varepsilon_{1R}$, $\;\varepsilon_{1U}$ are the relaxed and unrelaxed values of ε_1_.

According to the Debye model, ε_2R_ ≅0_,_ and so $\Gamma_{2}$_R_ ≅0 is $\Gamma_{2}$ = $\frac{\varepsilon_{2}}{(\varepsilon_{1}-1{)}^{2}+\varepsilon_{2}^{2}}$

For Equations (48) and (49), the condition (43) will be satisfied if the following are obtained:

(53) $$\frac{{(a+bx}^{2})(1+x^{2})}{b^{2}{x^{4}+x}^{2}\left(a^{2}+b^{2}\right)+a^{2}}=\frac{x(a-b)(1+x^{2})}{b^{2}{x^{4}+x}^{2}\left(a^{2}+b^{2}\right)+a^{2}}$$

from which, taking into account Equations (50)–(52), the following are obtained:

(54) $$\omega =\frac{1}{\sigma }\sqrt{\frac{\varepsilon_{1R}-1}{\varepsilon_{1R}+1-2\varepsilon_{1U}}}$$

This is the solution of Equation (43) in the Debye approximation. Equation (54) is a real solution (being $\varepsilon_{1R}>1)\;if$ the following equation is satisfied:

(55) $$\varepsilon_{1R}+1>{2\varepsilon }_{1U}$$

As can be seen in Equation (54), the solution of Equation (43) (when it exists) depends on σ, ε_1R_, ε_1U_. This means that, at every measure in which there is even a slight difference between ${\;\varepsilon }_{1R}$ and ε_1U_, it will be possible to find a ω value for which $a^{\left(0,0\right)}=a^{\left(1,1\right)}$. It should be emphasized that, as can be seen in Equation (54), there is only one ω_i_ value at each measurement. Generalizing and correlating Equation (54) at each measure can be written as follows:

(56) $$\omega_{i}=\frac{1}{\sigma_{i}}\sqrt{\frac{\varepsilon_{1R}^{\left(i\right)}-1}{\varepsilon_{1R}^{\left(i\right)}+1-2\varepsilon_{1U}^{\left(i\right)}}}$$

In this case, Equation (55) is as follows:

(57) $$\varepsilon_{1R}^{\left(i\right)}+1>2\varepsilon_{1U}^{\left(i\right)}$$

ωi increasing or decreasing values can only be determined experimentally over time. Obviously, σ, ε1R, and ε1U will also depend on time because they are related to each measurement made to the passage of time. It will be written as follows:

ω_i_ = ω_i_(t_i_) (58)

that is

(59) $$\omega_{i}=\omega_{i}[\varepsilon_{1R}^{\left(i\right)}(t_{i}),\;\varepsilon_{1U}^{\left(i\right)}\left(t_{i}),\sigma_{i}(t_{i}\right)]={\tilde{\omega }}_{i}\left(t_{i}\right)$$

Because at each measurement, ω_i_ is the ω value for which ${\;a}^{\left(0,0\right)}=a^{\left(1,1\right)}$, ω_i_ can only be determined experimentally, with a fitting process. By substituting Equation (56) into Equation (47), we obtain P^(1)^ at the frequencies ω_i_. But substituting the explicit form of Equation (59) into Equation (47), we obtain P^(1)^, for which $a^{\left(0,0\right)}=a^{\left(1,1\right)}$ as a function of time; these results, obtained only by means of dielectric measurements, provide important information on the temporal evolution of a tumor.

## 3. Results

**Figure 1:**
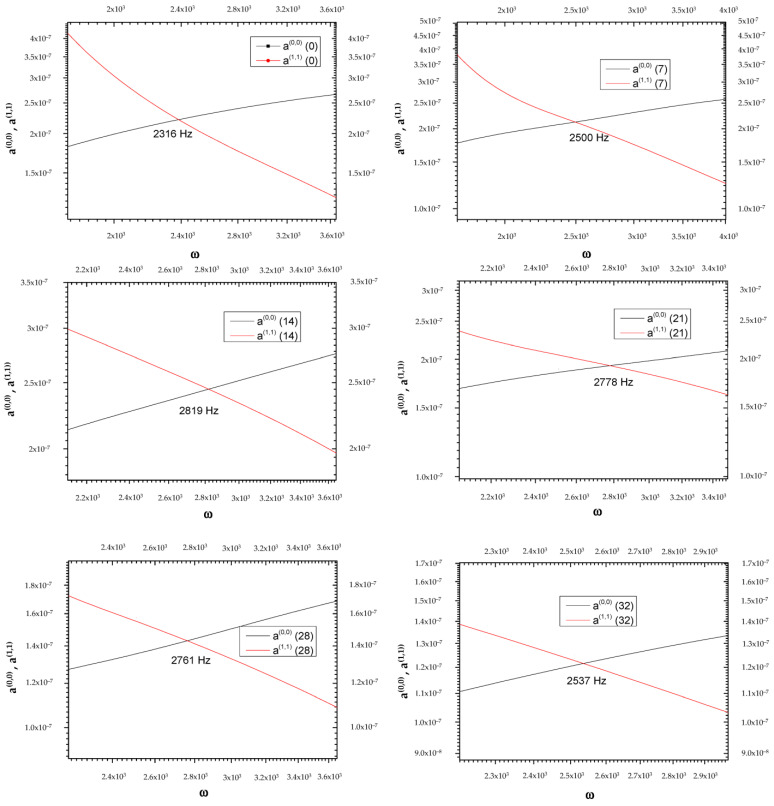
Graphical representation of frequencies at which the two curves, $a^{\left(0,0\right)}$ and $a^{\left(1,1\right)}$, intersect at the day variations.

**Figure 2:**
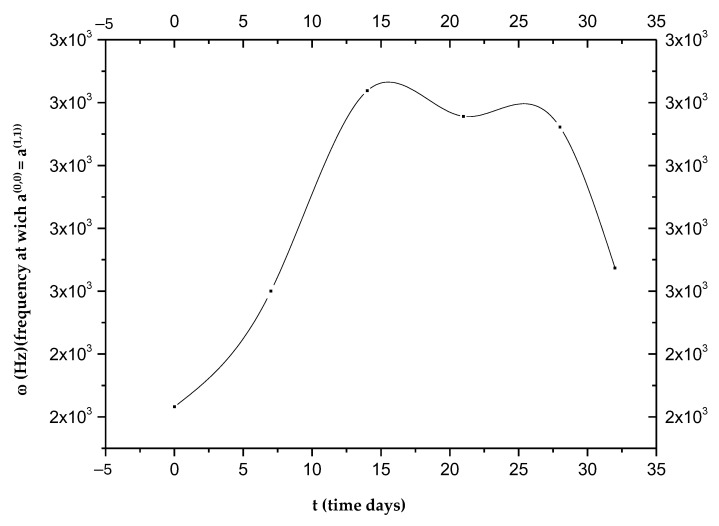
Frequencies at which the two curves, $a^{\left(0,0\right)}$ and $a^{\left(1,1\right)}$, intersect as a function of time.

The goal of the present investigation is the identification of a new path for the temporal study of tumor pathologies, although the limit of our research is the low number of experimental measurements (only six frequency values for which $a^{\left(0,0\right)}=a^{\left(1,1\right)}$). The two curves, $a^{\left(0,0\right)}$ and $a^{\left(1,1\right)}$, represented as a function of the frequency on days 0, 7, 14, 21, 28, and 32, intersect at frequencies that vary on the days considered (see Table 1, Figure 1 and Figure 2).

The low number of experimental measurements does not allow them to fit the data, so we cannot obtain the following curve:

ω = ω (t)(60)

As can be seen in Figure 2, each ω_i_ value does not correspond to a single t_i_; therefore, with the curve alone, it is not possible to establish a one-to-one correspondence between the frequency ω and the time elapsed from the beginning of the phenomenon attributed to intravasation. Figure 2 shows the presence of a maximum value around the 14th day, which defines two opposite trends, t > 14 and t < 14. This change indicates a turnaround of some biological phenomena that we will refer to as “phenomenon X”. It is possible to establish the time elapsed from the beginning of the invasion by measuring ω_i_ values. In detail, the curves of P^(0)^ and P^(1)^ as a function of frequency [23] allow us to establish whether the time elapsed from intravasation is before or after the 14th day; in fact, both curves change their concavity exactly on the 14th day. By detecting the value of ω_i_ in the two ranges, we can determine the day from the intravasation. Obviously, all data should be verified over a considerable number of cycles of measurements to irrefutably affirm the goodness of the idea presented in this study. For more details on the used mathematical formulas, see Appendix A.

**Figure 3:**
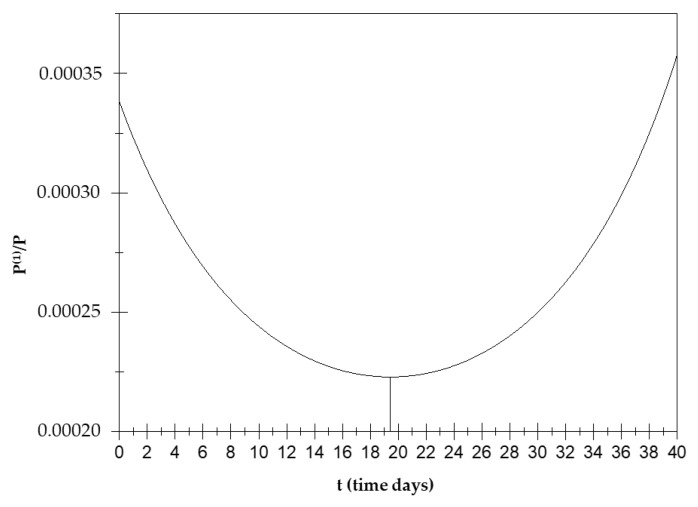
Orientation polarization trend $\frac{P^{\left(1\right)}}{P}$ over time.

Now, by way of illustration, it is supposed that the data represented in Figure 2 can be fitted with a parabola:

Figure 3 shows the orientation polarization trend over time with minimum value of $\frac{P^{\left(1\right)}}{P}$ for t_2_ = 19.4 days; therefore, $\frac{P^{\left(1\right)}}{P}$ is a decreasing function up to t_2_ (see Appendix A (A3)–(A6)).

This means that, when $a^{\left(0,0\right)}=a^{\left(1,1\right)}$, the orientation polarization has an increasing trend over time up to a minimum value around the 19th day and then decreases.

The displacement current is then calculated (that is, the temporal variation in the electric field in a system) $\frac{{dP}^{\left(1\right)}}{dt}$ for the frequencies in which $a^{\left(0,0\right)}=a^{\left(1,1\right)}$ (see Appendix A (A7)–(A15)).

(61) $$\frac{{dP}^{\left(1\right)}}{dt}=[\frac{\omega^{2}\sigma }{\left(1+\omega^{2}\sigma^{2}\right)}]P$$

**Figure 4:**
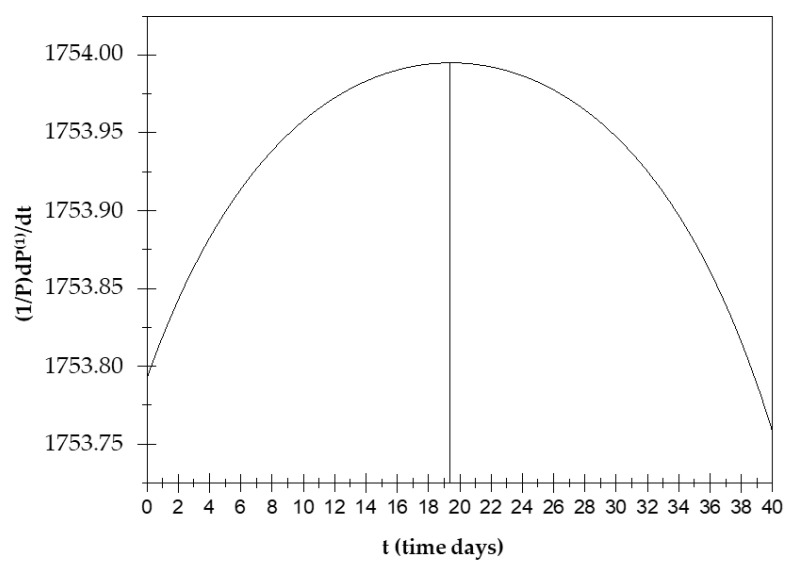
Displacement current trend over time.

At the points in which a^(0,0)^ = a^(1,1)^, the displacement current increases until about the 19th day and then decreases until the 32nd day (see Figure 4).

The study of this function is very important as displacement currents can be the cause of some biological phenomena, including changes in the conformational T-R states of hemoglobin. In the hemoglobin case, displacement current processes deactivate ionic pairs, which are a cause of stabilization of the T state [20]. In the present case, instead, the displacement currents contribute to the occurrence of phenomenon X due to orientation polarization. Due to this polarization, the orientation polarization and displacement currents exhibit opposite trends over time.

Then, we calculate E^(1)^; the E^(1)^ trend decreases until the 19th day and then increases until the 32nd (see Appendix A (A16)–(A21)). It is very important to underline the maximum or minimum values around the 19th day for all functions; this condition is attributable, from a physical point of view, to the presence of a field opposite the perturbing field, which has been explained by Farsaci et al. (2019) by means of the polarization of CTCs and activated platelets [21]. Here, we reiterate that the variation in $\frac{dP^{\left(1\right)}}{dt}$ is due to the field generated by the high number of negative charges that surround the CTCs and activated platelets, which oppose the perturbing field. We emphasize that all the figures shown in this paper refer to the frequencies for which $a^{\left(0,0\right)}=a^{\left(1,1\right)}$, and that these frequencies vary between 2300 and 2800 Hz (see Table 1); therefore, we are in a range of low frequencies. Next, the entropy production is calculated; it can be shown that, in this case, the following was determined [22]:

(62) $$\sigma^{\left(s\right)}=\frac{P_{0}^{2}}{Τ}\omega \Gamma_{2}\;\Gamma_{2}=\frac{(a-b)\omega \sigma }{a^{2}+b^{2}\omega^{2}\sigma^{2}}$$

It is easy to show that the entropy trend is also affected by time up to the 19th day: $\sigma^{\left(s\right)}$ increases until the 19th day and then decreases; thus, the disorder of the system increases until the 19th day, but then, the situation is reversed, and the system trend is towards an increase in order.

## 4. Discussion

### X Phenomena Cause: A Thermodynamic Explanation

The results obtained so far lead us to explain what happens around the 19th day, namely the “X phenomenon”, through the generation of displacement currents originating, in turn, from charge variations and, therefore, from dipole movements (see Appendix A (A22)–(A24)).

**Figure 5:**
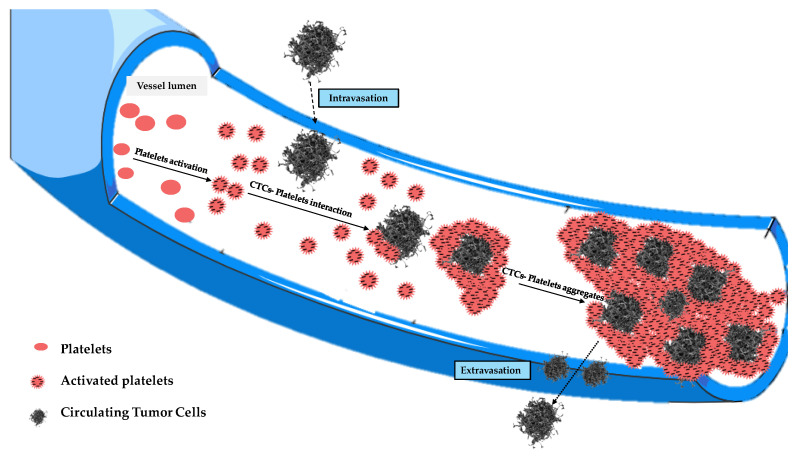
Evolution of tumor cell–platelet interplay during the tumor cells’ migration in the blood circulation.

It is good to remember that in physiological blood there are about 1.5 × 10^5^  ÷ 4 × 10^5^ platelets per mm^3^, each of which has dimensions of (2 ÷ 4) μm. It is well known that platelets are negatively charged, and they will tend to form aggregates in the blood, which are called clusters. Their number usually increases in the presence of CTCs, since a platelet–CTC interaction generally occurs after intravasation, with the formation of aggregates that contribute to protecting CTCs from immune surveillance, apoptosis, blood turbulence, and shear stress, ensuring tumor cell survival in the blood circulation (Figure 5). In fact, blood represents a very hostile environment for CTCs that can be easily destroyed by hydrodynamic forces in the blood circulation, which are often very strong in small vessels. This is where platelets come into play, which, although part of the body’s autoimmune defense, surround the cancer cells to form a sort of shell that is exploited by them to move within the blood circulation.

**Figure 6:**
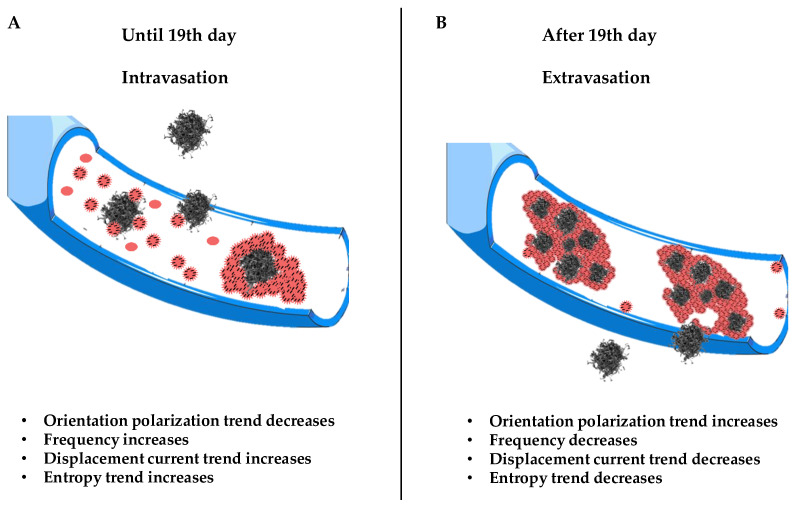
Schematic illustration of the X phenomenon. Section (**A**) shows the trigger of CTC dissemination in blood from 0 to 19th days. Section (**B**) shows the evolution of CTC invasion in blood and the extravasation process.

Those aggregates might assume considerable dimensions (up to about 50 μm) during their motion, having a high chance of remaining trapped in the pulmonary capillaries [23,24,25,26]. Then, only a small percentage of CTCs manage to remain viable, and only for a short period of time. The adhesion of these cells to the inner walls of the arterioles and capillaries of certain tissues provides the first foothold within the tissues themselves, and the continuation of dissemination through the process of extravasation is a complicated, multi-step process. Adhesion of CTCs onto the capillary endothelium causes the formation of a micro-thrombus that will be dissolved within a day by the proteases responsible for dissolving clots. Therefore, less than 1% of CTCs will be able to metastasize after extravasation [24]. Why do displacement currents increase in the early days? It is known that the different concentrations of charges (due to chemical elements) cause the surface of the outer membrane to have a relatively high positive charge [27]. On the other hand, the outermost negative zone of the calyx with constant negativity makes each zone like a negatively charged body [28]; the outermost negative zone is separated from the positive surface of the cell membrane by a dielectric phospholipid bilayer that separates the extracellular space and the cytoplasm. Since the platelets are negatively charged and about 2–4 μm in size, they can easily envelop the positive cell membrane, which already contains many dipolar molecules [29,30,31,32,33]. This can trigger the production of an additional number of dipoles beyond those already existing. This process causes more and more dipoles to form because, over time, the number of CTCs will increase. This explains the trigger of displacement currents over time, up to the 19th day (Figure 6). On the other hand, the decrease in P^(1)^ over time is natural because greater times correspond to greater ω_c_, and the CTCs bound to platelets are very large; as the frequencies increase (up to the 19th day), the mean dipole moment decreases (therefore P^(1)^). Ultimately, we can state that the cause of the increase in displacement currents is due to the production of platelets. At each measurement, starting from day “0”, the frequency at which a^(0,0)^ = a^(1,1)^ increases with time, until it reaches its maximum of ω ≅ 2800 Hz on the 19th day, approximately. The displacement current also increases due to the orientation polarization calculated at the frequency for which a^(0,0)^ = a^(1,1)^ results.

We state that field E^(1)^ = E^(eq)^ + ${\underline{E}}_{p}^{\left(1\right)}$, which opposes the initial perturbation due only to the polarization by deformation since E^(eq)^ = a^(0,0)^ P^(0)^. We are not stating that there is no orientation polarization; in fact, P^(1)^ for ω such that a^(0,0)^ = a^(1,1)^ is different from zero. Through the equation ${\underline{E}}_{\underline{p}}^{\left(1\right)}$ = (a^(0,0)^ − a^(1,1)^) P^(1)^ = 0, we only state that this polarization generates a field that is null. The question is, what happens in the blood at the frequency for which ${\underline{E}}_{\underline{p}}^{\left(1\right)}=0$?

It is clear that what makes ${\underline{E}}_{\underline{p}}^{\left(1\right)}=0$ possible are the displacement currents associated with the polarization by orientation. At every measurement, and therefore over time, there is a frequency for which ${\underline{E}}_{\underline{p}}^{\left(1\right)}=0$; then, there is a frequency for which the field E^(eq)^ equals the field $\frac{1}{L^{\left(1,1\right)}}\;\frac{dP^{\left(1\right)}}{dt}$ generated by the displacement currents. This means that the field associated with the polarization by deformation and that associated with the displacement currents are equal; this form of equilibrium always shifts to higher frequencies as time passes until the 19th day. Since we are at low frequencies, what oscillates (rotates) are relatively large objects. These can only be the CTC–platelet aggregates that rotate and deform, generating two equal and opposite fields. The ever-increasing number means that this equality occurs at ever higher frequencies, as there will be more and more CTCs without platelets entering and helping to generate two fields; with CTCs being smaller than CTC–platelet aggregates, they will oscillate at higher frequencies. There is an intermediate range of frequencies in which CTC–platelet aggregates and CTCs alone oscillate and rotate simultaneously. Obviously, this range is the subset of frequencies for which a^(0,0)^ = a^(1,1)^ or ${\underline{E}}_{\underline{p}}^{\left(1\right)}=0$. This also explains why at high frequencies, $\frac{dP^{\left(1\right)}}{dt}$ decreases over time; this is because fewer and fewer CTC–platelet aggregates oscillate. After the 19th day, there is a reversal trend for both displacement currents $\frac{dP^{\left(1\right)}}{dt}$ and P^(1)^: $\frac{dP^{\left(1\right)}}{dt}$ decreases and P^(1)^ increases because fewer CTCs enter, and therefore, there will be fewer CTC–platelet aggregates over time. It should be noted that the frequencies at which a^(0,0)^ = a^(1,1)^ after the 19th day decrease; therefore, when the CTC–platelet aggregates are very large, the frequency decreases (until the 32nd day) and the average dipole moment increases. Figure 4 shows a decrease in displacement currents as there is an ever-smaller number of charges of the CTCs and platelets that are deposited on the CTCs. We could ascribe this inversion to a subsequent spontaneous attenuation in the CTC release, which is likely due to a dormancy of the primary tumor, correlated with a lack of angiogenesis, which restricts its ability to grow beyond a certain size. We conclude by stating that these trend interactions are caused by a number of entering CTCs and several platelets being generated (Figure 6).

The “X phenomenon” that we would like to elucidate can be identified, ultimately, as the turnaround in the number of CTCs after the 19th day. This explanation was possible since we have identified in the displacement currents $\frac{dP^{\left(1\right)}}{dt}$ the “cause” for which it results a^(0,0)^ = a^(1,1)^ and ${\underline{E}}_{\underline{p}}^{\left(1\right)}=0.$

## 5. Conclusions

The dielectric study of blood, supported by the developed thermodynamic approach, afforded completely new and interesting results. In fact, the finding of a one-to-one correspondence between the frequencies in which a^(0,0)^ = a^(1,1)^ and the time elapsed since tumor inoculation allows for establishing the tumor progression time by measuring the frequency with which a^(0,0)^ = a^(1,1)^. The developed algorithm finds practical application in the construction of a database of dielectric measurements of blood for various types of tumors, for which there is a frequency at which equality is verified. Although the study of this topic is still under development, it could lay the groundwork for a new diagnostic approach. Moreover, the present investigation analyzed, for the first time, the role of displacement currents to explain otherwise unexplainable phenomena, i.e., the decrease over time of cancer cells in the blood. In fact, most of the stages of tumor formation are known in detail, but knowledge of the process of invasion and metastasis is still very incomplete. The mass of neoplastic cells of a malignant tumor, through the release of specific proteases, manages to overcome the basement membrane, cross the walls, and enter the lumen of the blood vessels. Once the cells penetrate the vessel, they can transmigrate to other districts. Within a few days, CTCs proliferate and make their way through the capillary basement membrane, invading the surrounding parenchyma of tissue far from the primary tumor that is apparently normal [34]. These findings can be explained by a model associated with the X phenomenon in which the invasion and number of cancer cells in the blood increases in the first two weeks until it reaches a peak around the nineteenth day, after which the concentration of CTCs decreases again, assuming the initiation of the extravasation process. The results obtained in this study highlight the thermodynamic approach in the early stage of cancer study. In detail, the application of our thermodynamic theories to intravasation and extravasation phenomena allowed us to follow the evolution of cancer in the blood from the very first days. This model is based on changes in the dielectric properties of blood following the dissemination of cancer cells, which paves the way for the initiation of new study techniques and for the development of biosensors for the early detection of cancer. This study gives medicine research a new point of view for new therapeutic perspectives, with the aim of reducing cell migration and cancer expansion.

## Figures and Tables

**Table 1:** Frequency on days in which the two curves a^(0,0)^ = a^(1,1)^ intersect.

t_i_ Days	w_i_ Hertz
0	2316
7	2500
14	2819
21	2778
28	2761
32	2537

## Data Availability

The original contributions presented in this study are included in the article. Further inquiries can be directed to the corresponding author.

## Abbreviations

The following abbreviations are used in this manuscript:

CTCs	Circulating Tumor Cells
CTMs	Circulating Tumor Microemboli
CT	Computerized Tomography
MRI	Magnetic Resonance Imaging
PET	Positron Emission Tomography
TTM	Thermodynamic Tumor Matrix

## Appendix A

The data of frequencies at which the two curves a^(0,0)^ and a^(1,1)^ intersect as a function of time can be fitted with a parabola as follows:

(A1) $$\omega =\overset{ˇ}{a}t^{2}+\overset{ˇ}{b}t+\overset{ˇ}{c}$$

where

(A2) $$\overset{ˇ}{a}=\frac{1.4}{{giorni}^{2}*\;s}\;;\;\overset{ˇ}{b}=\frac{54.4}{giorni*\;s}\;;\;\overset{ˇ}{c}=\frac{2277}{s}$$

In Equation (A1), t and ω are expressed in days and in Hz, respectively. At each frequency ω, Equation (A1) gives two values of t; as previously stated, the uniqueness of t is obtained from the concavity of the P^(0)^ and P^(1)^ curves. The values of P^(1)^ decrease as ω (and therefore t) increases in accordance with Equation (47), assuming that Equation (A1) is a continuous function of t, defined by 0 ≤t≤32 days. By substituting Equation (A1) into Equation (47), we can obtain the following:

(A3) $$\frac{P^{\left(1\right)}}{P}=y_{1}=\frac{1}{1+(-\overset{ˇ}{a}t^{2}+\overset{ˇ}{b}t+\overset{ˇ}{c}{)}^{2}\sigma^{2}}$$

Here, we assume that Equation (A1) is a solution of Equation (43). P^(1)^ is normalized for P so as to study the trend of $\frac{P^{\left(1\right)}}{P}$, which we denote by y_1_. We see the maximum and minimum because this is what we are interested in studying, and we study the sign of the numerator of the derivative of y_1_ with respect to time (the denominator is certainly positive as to the square).

(A4) $$\mathrm{num}\;(\frac{dy_{1}}{dt})=-[2\left(-\overset{ˇ}{a}t^{2}+\overset{ˇ}{b}t+\overset{ˇ}{c}\right)\left(-2\overset{ˇ}{a}t+\overset{ˇ}{b}\right)\sigma^{2}]>0$$

Based on the above equation, the following is obtained:

$$\left\{\begin{array}{lll} \;-\overset{ˇ}{a}t^{2}+\overset{ˇ}{b}t+\overset{ˇ}{c}<0 & \;t<t_{1}=\frac{b+\sqrt{\overset{ˇ}{b^{2}}+4\overset{ˇ}{a}\;\overset{ˇ}{c}}}{2\overset{ˇ}{a}} & \;(A5)\\ -2\overset{ˇ}{a}t+\overset{ˇ}{b}>0 & \;t<t_{2}=\frac{\overset{ˇ}{b}}{2\overset{ˇ}{a}} & \;(A6) \end{array}\right.$$

By substituting Equation (A2) into Equations (A4) and (A5), the following is obtained:

$$\left\{\begin{array}{c} t_{1}=64.1\;\mathrm{days}\;\\ t_{2}=19.4\;\mathrm{days}\;\; \end{array}\right.$$

The value of t_1_ is out of the definition range, and therefore, we will consider t_1_ < 32 days. There will be a minimum for y_1_ = $\frac{P^{\left(1\right)}}{P}$ in t = 19.4 days. Figure 3 shows a minimum value of $\frac{P^{\left(1\right)}}{P}$ for t_2_ = 19.4 days; therefore $\frac{P^{\left(1\right)}}{P}$ is a decreasing function up to t_2_.

When $a^{\left(0,0\right)}=a^{\left(1,1\right)}$, the orientation polarization has an increasing trend over time up to a minimum value around the 19th day and then decreases.

We then calculate $\frac{{dP}^{\left(1\right)}}{dt}$ for the frequencies in which $a^{\left(0,0\right)}=a^{\left(1,1\right)}$. P^(0)^ and P^(1)^ can be associated with a so-called affine electric field E^(1)^ given by the following [19]:

E^(1)^ = a^(0,0)^
P^(0)^ + (a^(0,0)^ − a^(1,1)^) P^(1)^
(A7)

or

(A8) $${\underline{E}}^{\left(1\right)}={\underline{E}}^{\left(\mathrm{eq}\right)}+{\underline{E}}_{\underline{p}}^{\left(1\right)}$$

This is the field that is opposed to the perturbation. E^(eq)^ = a^(0,0)^ P^(0)^ is the equilibrium electric field [19]. Since we consider frequencies for which $a^{\left(0,0\right)}=a^{\left(1,1\right)}$, the last equation becomes the following:

E^(1)^ = E^(eq)^ (A9)

Moreover, the following phenomenological equation is valid [19]:

(A10) $$\frac{{dP}^{\left(1\right)}}{dt}=L^{\left(1,1\right)}{\underline{E}}^{\left(1\right)}$$

and in our case, the equation becomes the following:

(A11) $$\frac{{dP}^{\left(1\right)}}{dt}=L^{\left(1,1\right)}{\underline{E}}^{\left(eq\right)}$$

It can be shown that ${\underline{E}}^{\left(eq\right)}$ = $\Gamma_{1}\;P$ and Equation (A11) becomes the following [19]:

(A12) $$\frac{{dP}^{\left(1\right)}}{dt}=L^{\left(1,1\right)}\Gamma_{1}\;P$$

Moreover, it can be shown that the L^(1,1)^ value is obtained from L^(1,1)^ = $\frac{1}{a^{\left(1,1\right)}\sigma \;}$, considering Equation (44):

(A13) $$L^{\left(1,1\right)}=\frac{1}{\Gamma_{2}^{\left(1\right)}(\frac{1+\omega^{2}\sigma^{2}}{\omega \sigma })\sigma \;}=\frac{\omega }{\Gamma_{2}^{\left(1\right)}(1+\omega^{2}\sigma^{2})\;}$$

which is substituted into Equation (A12):

(A14) $$\frac{{dP}^{\left(1\right)}}{dt}=\frac{\Gamma_{1}}{\Gamma_{2}^{\left(1\right)}}\;(\frac{\omega }{1+\omega^{2}\sigma^{2}\;\;})P$$

For Equation (43), this last result is obtained:

(A15) $$\frac{{dP}^{\left(1\right)}}{dt}=[\frac{\omega^{2}\sigma }{\left(1+\omega^{2}\sigma^{2}\right)\;}]$$

Due to the polarization, the orientation polarization and displacement currents exhibit opposite trends over time. We calculate E^(1)^, and in this case, the following result is obtained:

(A16) $$E^{\left(1\right)}=E^{\left(\mathrm{eq}\right)}=\Gamma_{1}P$$

We consider only $\Gamma_{1}$ because we normalize for P. Using the Debye approximation, the denominator of Equation (48) can be written as follows:

(A17) $$\left(a^{2}+b^{2}\omega^{2}\sigma^{2})(1+\omega^{2}\sigma^{2}\right)$$

Therefore, Equation (48) is as follows:

(A18) $$\Gamma_{1}=\frac{a+b\omega^{2}\sigma^{2}}{a^{2}+b^{2}\omega^{2}\sigma^{2}}$$

For Equation (A16), by studying the trend of $\Gamma_{1}$ we will determine the trend of $\frac{E^{\left(1\right)}}{P}$. As demonstrated above, we studied only the numerator of the derivative $\frac{d\Gamma_{1}}{dt}$ after substituting Equation (A1) into (A18). The result is as follows:

$$\Gamma_{1}=\frac{a+b(-\overset{ˇ}{a}t^{2}+\overset{ˇ}{b}t+\overset{ˇ}{c}{)}^{2}\;\sigma^{2}}{a^{2}+b^{2}(-\overset{ˇ}{a}t^{2}+\overset{ˇ}{b}t+\overset{ˇ}{c}{)}^{2}\;\sigma^{2}}$$

$$\mathrm{num}\;\frac{d\Gamma_{1}}{dt}\;=-2ab^{2}\sigma^{2}\left(-\overset{ˇ}{a}t^{2}+\overset{ˇ}{b}t+\overset{ˇ}{c}\right)\left(-2\overset{ˇ}{a}t+\overset{ˇ}{b}\right)>0$$

(A19) $$\begin{array}{c} \left(-\overset{ˇ}{a}t^{2}+\overset{ˇ}{b}t+\overset{ˇ}{c}\right)<0\\ -2\overset{ˇ}{a}t+\overset{ˇ}{b}>0 \end{array}$$

This system is equal to the system of Equations (A4) and (A5); by solving it we see that, in time, the trend of E^(1)^ is in opposition to the displacement currents $\frac{dP^{\left(1\right)}}{dt}$; the E^(1)^ trend decreases until the 19th day and then increases until the 32nd. Next, the entropy production is calculated; it can be shown that, in this case, the following result is obtained:

(A20) $$\sigma^{\left(s\right)}=\frac{P_{0}^{2}}{Τ}\left[\frac{\omega^{2}}{L^{\left(1,1\right)}\;}\left(1-\frac{\Gamma_{1}}{a^{\left(0,0\right)}}\right)\right]{cos}^{2}\omega t=\;\frac{P_{0}^{2}}{Τ}\left[\omega \Gamma_{2}^{\left(1\right)}\left(1+\omega^{2}\sigma^{2}\right)\left(\frac{{\Gamma_{2}^{\left(1\right)}+\Gamma_{2}^{\left(1\right)}\omega^{2}\sigma^{2}-\Gamma }_{1}\omega \sigma }{\Gamma_{2}^{\left(1\right)}(1+\omega^{2}\sigma^{2})}\right)\right]{cos}^{2}\omega t=$$

(A21) $$\frac{P_{0\omega }^{2}}{Τ}[\Gamma_{2}^{\left(1\right)}+\Gamma_{2}^{\left(1\right)}\omega^{2}\sigma^{2}-\Gamma_{2}^{\left(1\right)}\omega^{2}\sigma^{2}]{cos}^{2}\omega t\;\sigma^{\left(s\right)}=\frac{P_{0}^{2}}{Τ}\omega \Gamma_{2}\;\Gamma_{2}=\frac{(a-b)\omega \sigma }{a^{2}+b^{2}\omega^{2}\sigma^{2}}$$

To better clarify the starting hypothesis, the concept of polarization is briefly recalled. Let r_q_ and r_−q_ be the position vectors of the charges +q and −q, respectively, with respect to a point O (pole). This is called the dipole moment with respect to O, which is indicated with P_dip_, the vector, from which we obtain the following equation:

P_dip_ = q r_q_ + (−q) r_−q_ (A22)

The following vector P is the dipole moment per volume unit (or polarization vector), if n dipoles are contained in a volume V:

(A23) $$\underline{P}=\sum_{i}^{n}\frac{\;P_{i}r_{i(q)}+(-q)r_{i(-q)}}{V}$$

That is, the dipole moment per unit of volume is the vector sum of the n dipoles divided by the volume V that contains them.

Therefore, we rewrite Equation (A11) in the following form:

(A24) $$\frac{1}{L^{\left(1,1\right)}}\;\frac{dP^{\left(1\right)}}{dt}=E^{\left(\mathrm{eq}\right)}$$

and remembering that, in this case, ${\underline{E}}_{\underline{p}}^{\left(1\right)}$ = (a^(0,0)^ − a^(1,1)^) P^(1)^ = 0, we state that the field E^(1)^ = E^(eq) +^ ${\underline{E}}_{p}^{\left(1\right)},$ which opposes the initial perturbation, is due only to the polarization by deformation since E^(eq)^ = a^(0,0)^ P^(0)^. We are not stating that there is no orientation polarization; in fact, P^(1)^ for ω such that a^(0,0)^ = a^(1,1)^ is different from zero. Through the equation ${\underline{E}}_{\underline{p}}^{\left(1\right)}$ = (a^(0,0)^ − a^(1,1)^) P^(1)^ = 0, we only state that this polarization generates a field that is null. The question is, what happens in the blood at the frequency for which ${\underline{E}}_{\underline{p}}^{\left(1\right)}=0$?

Since Equation (A10) can be rewritten $as\;\frac{1}{L^{\left(1,1\right)}}\;\frac{dP^{\left(1\right)}}{dt}$ − E^(eq)^ = ${\underline{E}}_{\underline{p}}^{\left(1\right)}$, what makes ${\underline{E}}_{\underline{p}}^{\left(1\right)}=0$ possible are the displacement currents associated with the polarization by orientation.

## Appendix B

### Glossary

S Entropy S = S(U|P)

U Internal Energy

P Polarization vector P = P^(0)^ + P^(1)^

P^(0)^ and P^(1)^ are two parts of the polarization vector P related to polarization by deformation and orientation, respectively

E is the electric field that appears in Maxwell’s equations, E = E^(eq)^ + E^(ir)^

E^(ir)^ is the irreversible electric field vector

E^(eq)^ is the equilibrium electric field associated with the deformation polarization P^(0)^

E^(eq)^ =a^(0,0)^ P^(0)^

E^(1)^ is the affine electric field associated with the variation in S with respect to P^(1)^

T is the temperature assumed as constant

a^(0,0)^ and a^(1,1)^ are State Coefficients

L^(0,0)^ and L^(1,1)^ are phenomenological coefficients

${E\;}_{p}^{\left(1\right)}$ is the field associated with the orientation polarization P^(1)^ given by (a^(0,0)^ = a^(1,1)^)

ω is the frequency

ε_(ω)_ is the complex dielectric constant

ε_0_ is the dielectric constant of vacuum

ε_1_ and ε_2_ are the real and imaginary parts of ε_(ω)_

dP^(1)^/dt represents the displacement current caused by the orientation polarization effects

s^(s)^ is the entropy production for dielectric relaxation phenomena

E_0_(ω) is the amplitude of the electric field

Φ(ω) is the phase lag between P and E

Γ1(ω) and Γ2(ω) are the storage and loss dielectric moduli relating to non-dissipative and dissipative phenomena, respectively

δ is the relaxation time

Γ_2R_ is the relaxed value of Γ_2_

(TTM) Thermodynamic Tumor Matrix

ε^′^ and ε^″^ describe the “sum” of all dissipative phenomena

${\;\Gamma }_{1}$ and $\Gamma_{2}$ are dielectric moduli corresponding to the ω values at which the curves a^(0,0)^ and a^(1,1)^ cross

$\varepsilon_{1R}$, $\;\varepsilon_{1U}$ are the relaxed and unrelaxed values of ε_1_

$\frac{P^{\left(1\right)}}{P}$ is the orientation polarization trend over time

r_q_ and r_−q_ are the position vectors of the charges +q and −q, respectively

P_dip_ is the dipole moment with respect to O

P is the dipole moment per volume unit (or polarization vector)
